# Supplementary material for: Diverse and complex muscle spindle afferent firing properties emerge from multiscale muscle mechanics
Source: eLife. 2020 Dec 28;9:e55177. doi: 10.7554/eLife.55177 (PMC7769569; doi:10.7554/eLife.55177)
Supplement: Supplementary file 1. — These parameters did not change in any simulation presented in this study. When two values are presented, they represent the respective values for the dynamic and static fibers. [file elife-55177-supp1.docx]

| Parameter | Value (dynamic, static fiber) | Units | Description |
| --- | --- | --- | --- |
| $k_{cb}$ | 1 | mN m^-1^ | Unit cross-bridge stiffness |
| $x_{ps}$ | 2.5 | nm | Unit power stroke distance |
| $L_{thick filament}$ | 815 | nm | Length of thick filament |
| $L_{thin filament}$ | 1120 | nm | Length of thin filament |
| $L_{bare zone}$ | 80 | nm | Length of bare zone |
| $c_{filament}$ | 0.5 | - | Filament compliance factor |
| $\rho_{cb}$ | 6.9 $\times$10^16^ | m^-2^ | Cross-bridge number density |
| $l_{0}$ | 1050, 1200 | nm | Passive force reference length |
| $k_{pas}$ | 100, 250 | N m^-2^ nm^-1^ | Passive force linear stiffness |

**Supplementary File 1**

Constant parameters used in both dynamic and static intrafusal muscle fiber models. These parameters did not change in any simulation presented in this study. When two values are presented, they represent the respective values for the dynamic and static fibers.
